# Challenges of students and residents of human medicine in the first four months of the fight against the Covid-19 pandemic – Implications for future waves and scenarios

**DOI:** 10.1186/s12909-021-02962-8

**Published:** 2021-10-30

**Authors:** Benny Wohlfarth, Beat Gloor, Wolf E. Hautz

**Affiliations:** 1 Department of Visceral Surgery and Medicine, lnselspital, Bern University Hospital, University of Bern, Freiburgstrasse 18, 3010 Bern, Switzerland; 2 Department of Emergency Medicine, lnselspital, Bern University Hospital, University of Bern, Freiburgstrasse 18, 3010 Bern, Switzerland

## Abstract

**Introduction:**

In the fight against the Covid-19 pandemic, medical students and residents are expected to adapt and contribute in a healthcare environment characterized by ever-changing measures and policies. The aim of this narrative review is to provide a summary of the literature that addresses the challenges of students and residents of human medicine in the first 4 months of the fight against the Covid-19 pandemic in order to identify gaps and find implications for improvement within the current situation and for potential future scenarios.

**Methods:**

We performed a systematic literature search and content analysis (CA) of articles available in English language that address the challenges of students and residents of human medicine in the first 4 months of the fight against the Covid-19 pandemic.

**Results:**

We retrieved 82 articles from a wide range of journals, professional backgrounds and countries. CA identified five recurring subgroup topics: “faculty preparation”, «uncertainties and mental health», «clinical knowledge», «rights and obligations» and «(self-) support and supply». Within these subgroups the main concerns of (re-)deployment, interruption of training and career, safety issues, transmission of disease, and restricted social interaction were identified as potential stressors that hold a risk for fatigue, loss of morale and burnout.

**Discussion:**

Students and residents are willing and able to participate in the fight against Covid-19 when provided with appropriate deployment, legal guidance, safety measures, clinical knowledge, thorough supervision, social integration and mental health support. Preceding interviews to decide on reasonable voluntary deployment, the use of new technology and frequent feedback communication with faculties, educators and policymakers can further help with a successful and sustainable integration of students and residents in the fight against the pandemic.

**Conclusion:**

It is critical that faculties, educators and policymakers have a thorough understanding of the needs and concerns of medical trainees during pandemic times. Leaders should facilitate close communication with students and residents, value their intrinsic creativeness and regularly evaluate their needs in regards to deployment, knowledge aspects, safety measures, legal concerns and overall well-being.

## Background

The corona virus disease 2019 (Covid-19) due to Severe Acute Respiratory Syndrome Corona Virus 2 (SARS-CoV-2) evolved into an infectious disease of pandemic proportion [1]. Medical students and residents around the globe have been expected to adapt and contribute in a healthcare system with measures and policies changing by the hour. Reports regarding the preparation of those in training have been scarce in the early days of the pandemic and lacking overarching insights.

While there is an abundance of information for those in training from previous epidemic and pandemic scenarios [2, 3], the applicability of such information to the current pandemic is questionable. The last pandemic of similar proportions, the Spanish flu [4], occurred around the end of the life of William Osler. Modern medical education was still in its infancy and the setting, compared to today’s globalized life and available technology, differed significantly, holding only sparse implications for today’s scenario.

The aim of this narrative review is to provide a summary of the literature that addresses the challenges of students and residents of human medicine in the first 4 months of the fight against the Covid-19 pandemic in order to identify gaps and find implications for improvement within the current situation and for potential future scenarios.

## Methods

We retrieved articles written in English language that described the challenges of students and residents of human medicine in the first 4 months of the fight against the Covid-19 pandemic. With Wuhan Health Authorities initially reporting the first cases of pneumonia (of then unknown etiology) to the World Health Organization (WHO) on December 31, 2019 [5], we set the timeframe for the literature search from January 1, 2020 to April 30, 2020.

The main search was performed via PubMed. Additionally, we retrieved articles via SARS-CoV2-references [6], a publicly available search engine from the University of Bern, Switzerland, originally aimed at collecting Zika-Virus articles that was adapted for the Covid-19 pandemic. The last search update was performed on May 23, 2020 to include articles published within our defined timeframe that had a delayed entrance into the aforementioned databases. We performed the search using the following MeSH terms and Boolean operators (exemplary for PubMed):

((Covid* AND Residen* OR Covid* AND Trainee* OR Covid* AND Student* OR Covid* AND Education*) OR (Corona* AND Residen* OR Corona* AND Trainee* OR Corona* AND Student* OR Corona* AND Education*)) AND (2020/01/01:2020/04/30[edat])

**Fig. 1:**
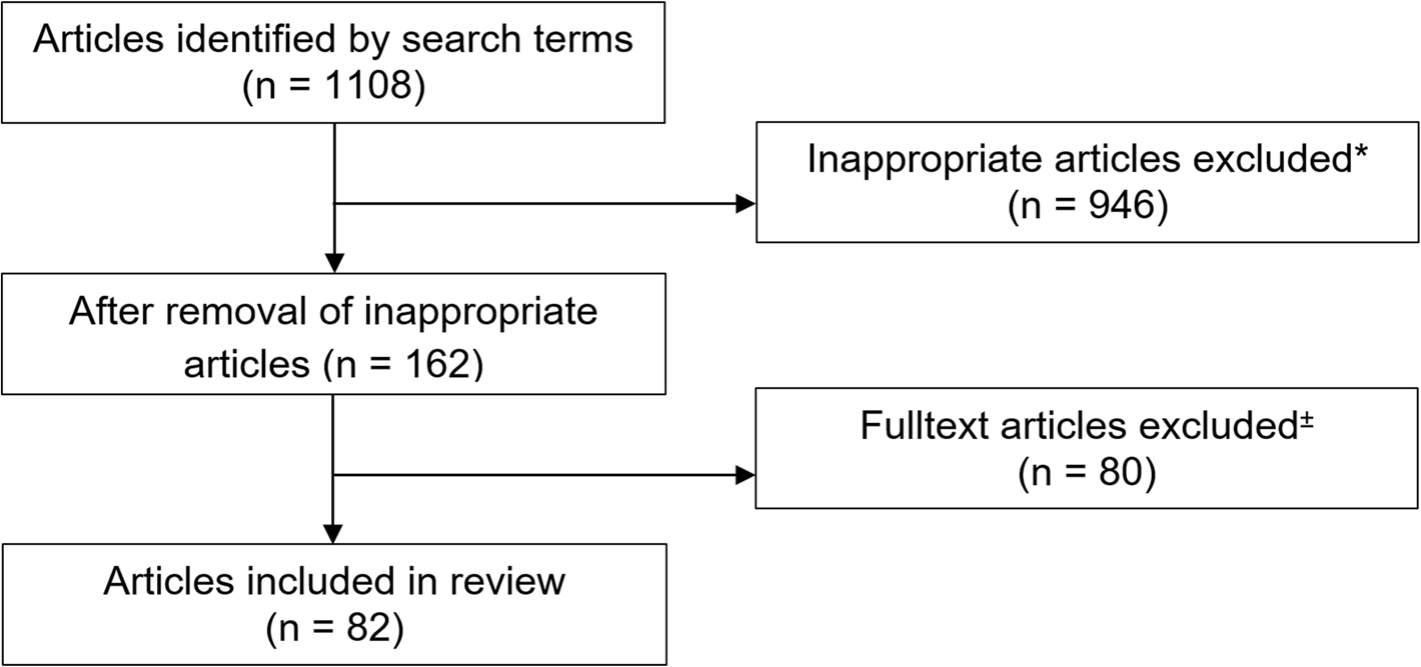
Process flowchart of article selection. ^*^inappropriate articles primarily resulted from articles in the pandemic that refer to 1. Residents and residency in terms of inhabitants or 2. Students, not in the sense of medical students or 3. Education, not in the sense of medical education. ^±^exclusion of articles solely dealing with adaptation of curricula

Up to May 23, 2020, we found 1108 articles using our search terms. After the elimination of inappropriate articles, we were left with 162 student- and resident-related articles. We then excluded 80 more articles that focused exclusively on curricula changes resulting from the Covid-19 pandemic (e.g. moving classes online [7]). The final pool consisted of 82 articles for review. [s. Figure 1].

The first author reviewed titles, abstracts, and full text of the articles for content analysis (CA) allocating the articles’ topics into five coded subgroups (s. Table 1) for synthesis and discussion. These subgroups were initially based on our own frontline experiences and iteratively refined in regards to insights gained from the review process:

- Faculty preparation
- Uncertainties and mental health
- Clinical knowledge
- Rights and obligations
- (Self-)support and supply

**Table 1:** Coded subgroups

Coded subgroup	No. of articles
Faculty preparation	(*n* = 34)
Uncertainties and mental health	(*n* = 24)
Clinical knowledge	(*n* = 10)
Rights and obligations	(*n* = 9)
(Self-)support and supply	(*n* = 5)
**Total**	**82**

Furthermore, metadata accompanying the articles was extracted to understand the overall perception and background of the topic, including publication rate, types of articles, professional background and country of residency of the first author (s. Table 2).

**Table 2:** Results of metadata analyzation

Professional background of first author	No. of articles	Country of residence of the first author	No. of articles	Type of article	No. of articles
Medical Student	(*n* = 10)	USA	(*n* = 43)	Letter	(*n* = 15)
Internal Medicine	(*n* = 8)	UK	(*n* = 14)	Commentary	(*n* = 14)
Medical Journalist	(*n* = 8)	Australia	(*n* = 2)	Study	(*n* = 12)
Orthopedic Surgery	(*n* = 7)	Canada	(*n* = 2)	News	(*n* = 7)
Neurosurgery	(*n* = 5)	Denmark	(*n* = 2)	Editorial	(n = 5)
Radiology	(*n* = 5)	Iran	(*n* = 2)	Perspectives	(n = 5)
Dermatology	(*n* = 4)	Singapore	(*n* = 2)	Correspondence	(*n* = 4)
Emergency Medicine	(*n* = 3)	South Korea	(*n* = 2)	Opinion	(*n* = 4)
Infectious Diseases	(*n* = 3)	UAE	(*n* = 2)	Career	(*n* = 3)
Head and Neck Surgery	(*n* = 3)	Others	(*n* = 11)	Innovation	(*n* = 2)
Surgery	(*n* = 3)	**Total**	**(*n*** **= 82)**	Medical Education Adaptations	(*n* = 2)
Anesthesiology	(*n* = 2)			Special Article	(*n* = 2)
Cardiology	(*n* = 2)			Others	(*n* = 7)
Psychiatry	(*n* = 2)			**Total**	**(*n*** **= 82)**
Psychology	(*n* = 2)				
Others	(*n* = 15)				
**Total**	**(*n*** **= 82)**				

## Results and findings

### General results and publication rate over time

Overall, 82 articles covered our topic. Of these articles, 70.7% (*n* = 58) articles solely and 29.3% (*n* = 24) articles partly (i.e., next to other topics) dealt with the challenges of students and residents of human medicine in the first 4 months of the fight against the Covid-19 pandemic.

**Fig. 2:**
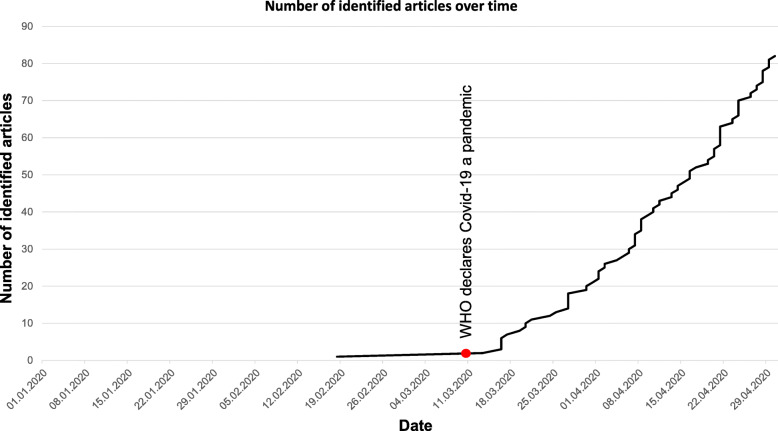
Number of identified articles over time

Prior to March 11, 2020, we identified only one appropriate article. From March 13, 2020 onwards, publication rate increased significantly [s. Figure 2].

### Coding results

Within the retrieved articles, we identified five recurrent subgroup topics in our CA [s. Table 1].

### Metadata analyzation of retrieved articles

We ranked the results from metadata analyzation according to professional background of the first author, country of residency of the first author and types of articles [s. Table 2].

### Coded subgroups

#### Faculty preparation

Initial countermeasures initiated by faculties ranged from single actions to complete workplace solutions [8–17]. Several departments started working in waves of alternating teams to avoid a complete breakdown in case of an outbreak amongst employees. While some were doing hospital service, others continued working from home, advising on inpatients remotely or doing telemedicine appointments on outpatients [13]. Further safety adaptations also included grouping of emergency department (ED) consultations from specialties whenever possible and strictly limiting the number of residents visiting Covid-19 positive patients during ward rounds [14].

To address the problem of surge capacity, students were deployed to information lines, epidemiological information services, childcare for frontline workers, laboratory work, screening clinics, contact tracing or even to the clinical setting. For the latter, considerations included everything from care of non-infected patients and low-acuity Covid-19 scenarios to introduction in ventilator therapy or nursing assistance [11, 18–24]. For clinical work, final year students were also given early registration, temporary licenses or allowed to work as physician assistants in several countries [11, 21, 23, 25–29].

With regards to clinical deployment, some individuals voiced safety concerns [30] while others considered clinically deployed students as an important measure to prevent personnel shortage [31]. Additionally, some educators view the pandemic as an opportunity to promote professional attributes such as altruism and solidarity within the profession [32, 33].

Residents from non-frontline specialties were often given the option to work, train or research from home or to apply for deployment on a voluntary basis [27, 34]. Regarding the latter, well-known concerns have been raised again, that, when compared to nurses and attendings, residents are often less likely to be trained adequately for potential mass casualties [35–37]. To assess the impact of deployment and to identify necessary actions, regular online meetings between students, residents and program directors have been initiated by faculties [12, 27, 38]. As further guidance, some departments shared their early guidelines for residency programs [39] and adapted article series from medical education journals where established to meet the unprecedented scenario [19, 40].

#### Uncertainties and mental health

In an early study looking at the impact of Covid-19 on students, 24.9% showed raised levels of anxiety. Namely, the stressors were related to economic struggles, impact on daily life and academic delays [41]. Furthermore, social distancing and isolation – inevitably linked to Covid-19 – are perceived as having a negative impact on well-being [42]. The disruption of training programs, reduced practical workload in a residents specialty, slowdown of research careers and non-voluntary deployment outside of one’s clinical competencies are further stressors raised to hold a risk for burnout, fatigue and loss of morale in trainees [43–46]. Residents were also concerned that the pandemic would adversely affect their completion of training and nearly half the respondents reported feeling anxious about their training future [47]. Also, more than half of the residents that were non-voluntarily employed in a workplace outside their usual scope of work showed higher levels of stress [11, 16, 47], while reports indicate that voluntarily deployed residents feel well-protected and trained [48].

Additionally, due to the potential risk of transmission, residents worried about the possibility of transmitting the disease to their families and patients [11, 42, 49–56] under the reported concerns of PPE shortage, vague instructions, limited testing capacities, frequently changing policies and the danger of mental exhaustion [54, 56]. Given that junior trainees are more likely to be deployed to areas of need than seniors [57], building on the aforementioned stressors, educators have raised concerns over the acceptable level of risk and who determines this level [56].

Medical students are also at potential risk of transmitting the disease and are aware of that [20, 21, 58]. Similar to residents’ concerns, PPE shortage was also a main stressor for students [58].

Social support seems to reduce levels of stress [41] and underline that staying connected in times of social distancing is particularly important [42]. It was brought up that the social aspect of discussing concerns with peers can also reduce anxiety and stress [33]. Early countermeasures to social isolation also included having residents participate in daily informal video conferences to promote social interaction with peers while practicing social distancing [14].

#### Clinical knowledge

Healthcare workers, including residents and students, revealed a varying knowledge about the pandemic in the beginning, with most in the need of improvement [59–62] and a few in good preparation [63, 64].

A lack of knowledge pertaining to Covid-19 was identified in several articles, ranging from basic uncertainties about the virus itself to complex scenarios like cardiopulmonary resuscitation (CPR) [56, 65] and even autopsy under infectious conditions [66]. Early approaches for knowledge improvement included senior residents establishing ‘Quick sheets’ about Covid-19. Initially set up to keep their peers up to date, these were quickly adopted by the whole department [67]. Radiologists were among the first to raise awareness for educating their trainees on the specific image findings in Covid-19 pneumonia [68]. Likewise, anesthesiologists reported early attempts to promote practical knowledge to trainees about the disease [69]. Focused training courses were established and could significantly increase knowledge about Covid-19 [70]. Here, instructor-led and video lesson based instruction on PPE showed no difference in quality [71]. Nevertheless, a major challenge surrounding the Covid-19 knowledge acquisition involved students’ and residents’ frequent use of social media as a source of information [59] with its potential risk of misinformation.

#### Rights and obligations

Amongst the main concerns were the legalities of training interruption, non-voluntary deployment and cancellation of exams with its subsequent consequences [20, 28, 53, 58, 72]. Some countries put postgraduate rotations on hold temporarily and residents remained in their current workplace with the possible exception of deployment to areas of need [73]. For undergraduate students, various organizations released statements to stop contact with patients to avoid unnecessary risks of infection. Correspondingly, students were excluded from clinical training in several countries, yet putting them into clinical practice in areas of need against Covid-19 was taken into account [28, 58, 74]. For a possible deployment to the frontline, medical associations demanded comprehensive introduction and supervision for students [25, 30, 75]. To minimize career disadvantages, several ideas were discussed, such as government-provided loan repayment or discounting on final-year tuitions [76].

In Canada, though final exams were postponed, the Royal College of Physicians and Surgeons affirmed that students graduating in 2020 will still be able to enter residency and to obtain a license [29]. In analogy to the acquisition of clinical knowledge the usage of social media with its potential risk of misinformation was also a complicating factor in legal orientation for students and resident, worsening insecurities [20].

#### (Self-)support and supply

Next to supportive measures aiming at clinical knowledge [67], one major topic was the self-assignment to appropriate tasks. Junior doctors began compiling support lists, with tasks that could be done from home and several universities and medical schools created groups to connect and assign students to appropriate roles within their national healthcare system and communities [28]. Also, students themselves formed response teams to connect peers to voluntary working opportunities in appropriate positions. This was done in close contact with administrations and faculties to monitor the evolving clinical needs and engage with students when necessary [77]. Furthermore, students formed various groups on social media to brainstorm ideas on how to support frontline workers in the fight against Covid-19 [21]. Practical examples also included various forms of support from childcare to meal preparation for medical professionals at the frontline [20].

Several students and residents reported that long working hours made it difficult to gather daily needed supplies. Therefore, peers began to set up supply boxes with sanitary products to help out each other correspondingly [78].

In one article, chief residents came up with a “Five Questions for Residency Leadership” guide to help adapting programs [79] under the current pandemic and students even got involved in constructing plastic face shields for frontline clinicians [46].

## Discussion

### General results and publication rate over time

Publications were scarce until early March 2020. After the WHO declaration on March 11, 2020 [1], we saw a profound increase in articles. We assume that the WHO declaration marked an important step for awareness and acted as an initiation for publication on the topic. Laudably, we found most publications to be open access.

Not surprisingly, only 14.6% (*n* = 12) of articles were studies while the majority represented short forms of communication, reporting timely from within the scenario.

### Most prolific background

Students are highly prolific in sharing their thoughts and experiences with Covid-19 and their input holds invaluable information for faculties, educators and policymakers. Furthermore, the participation of individuals with a background of internal medicine, emergency medicine, infectious disease and anesthesiology / intensive care holds invaluable implications for improvement. Even specialties who are less directly impacted by Covid-19 have also been very active. We do not only see this in the light of concern for deployment but also as an intrinsic motivation for solidarity within the profession.

### Most prolific country

Given our search filter for English language, articles were strongly dominated by Anglo-Saxon countries. To broaden the insight, a deeper involvement of other countries would have been desirable. Yet, we understand that priorities and resources were focused on frontline activities over the first 4 months.

### Most frequent article types

Here, short communication forms were more prevalent than studies or reviews. Due to the unprecedented situation, initial sharing of information was preferably done through quick communication forms like letters and commentaries, showing the eagerness for a rich discussion. Moreover, though the few studies we found were mostly survey-based, they must not be underestimated as they were able to deliver the first empirical information, particularly on the mindset and perception of the pandemic from students and residents.

### Coded subgroups

#### Faculty preparation

Owing to the large number of articles in this subgroup, it is safe to say that faculties recognize the importance of an adequate preparation of the youngest in training. Safety is a crucial factor, especially when it comes to clinical deployment. Students and residents must not be put in situations exceeding their competencies, should have adequate access to PPE and optimally be deployed on a voluntary basis [28, 30, 80]. Especially first year residents in deployment or new roles within their clinic need to be supervised appropriately [14, 25]. When deployed to the frontline, an adequate preparation and training must be provided for students and residents as it is for nurses and attendings [35–37]. Residents should have the chance to reduce physical movement and handle tasks remotely whenever possible. Therefore, their preparation, education and working routine (e.g., when on duty) should also involve new technology. Telemedicine, for example, can be used to combine patient care with education of residents and students [81] and simulation, due to its potential of lowering cognitive load, might help prepare residents for frontline deployment [82].

For an appropriate deployment, interviews can help to identify volunteers with the right intentions [32] and skills [28]. Within these interviews, aptitudes and motivation can also be recognized and give applicants the chance to fill in meaningful roles.

Lastly, to avoid shortage of staff, faculties must also take the potential lack of foreign exchange residents due to travel restrictions into account [83].

Students and residents are willing to participate, and faculties, educators and policymakers should recognize their concerns as well as their ideas. Thorough safety measures and supervision are mandatory for a successful deployment of students and residents. With the warranty of such measures, the current pandemic holds an invaluable chance for a successful integration and an important contribution for the professional identity formation of the youngest in training.

#### Uncertainties and mental health

Stress and anxiety arising from uncertainties in the current pandemic have a negative impact on students and residents [41, 84]. And though early studies occasionally have been criticized for not considering confounding factors [85], it is undeniable that Covid-19 has an impact on trainees’ mental health that needs to be addressed thoroughly to ensure their sustained successful integration in the workforce.

Deployment is a major uncertainty for many. And while deployment can also be perceived as an opportunity of personal growth and introspection [51], there is still room for improvement by giving trainees meaningful and adequate positions in the workforce. A crucial factor on how deployment is perceived lies in the form of selection and assignment. This is of great interest as raised stress levels in presumably non-voluntary deployment and training hold a risk for burnout [47]. For an optimal assignment, confidential interviews could help to assess issues that may have a negative impact on the trainee or their family (e.g., immunosuppression) and deployment should consider such information accordingly [44]. A sufficient Covid-19 testing capacity for staff, PPE and feasible shift schedules are further viable measures to reduce exposure and prevent burnout [50].

Social support, regular meetings and good teamwork also counteract the feeling of uncertainty [27, 48] and even physical home exercise routine with results being shared online among peers may be a way to strengthen social cohesion [12]. Also, the general possibility to consult mental health support [14] and raised awareness in psychiatrist for concerns and thoughts from frontline workers [42] seem sensible for an integral support. FAQs provided by journals and societies represented another reliable form of support for trainees [86]. From an educational point of view, competency based medical education can play a meaningful role to prepare students for healthcare crisis [49].

Leaders should stay in close contact with students and residents to provide information and emotional support [53]. Transparent communication, safety measures, teamwork culture, mental health support and a solid infrastructure for communication technology to secure remote social interaction are all approaches that can be implemented to diminish uncertainties, relieve stress and make the young workforce more resilient in unprecedented times.

#### Clinical knowledge

The knowledge about SARS-CoV-2 and Covid-19 is steadily evolving. To keep students and residents up to date, it is necessary to provide valid knowledge using widely distributed and accessible channels. With the vast majority of trainees’ being digital natives, most acquire their knowledge via online platforms and social media [59]. Faculties, educators and journals should foster the spread of valid clinical knowledge on social media and other digital solutions (e.g. online courses, chats and video conferences) as a mainstay for knowledge transfer. Fast distribution and relative inexpensiveness make such outlets even more attractive.

In addition, focused training courses online or offline seem a viable way to increase clinical and safety knowledge about Covid-19 [70, 71] . Local solutions like the aforementioned ‘Quick sheet’ may also be a fast and cost-efficient approach to provide Covid-19 related knowledge for students and residents.

#### Rights and obligations

Students and residents need clear information on their rights and obligations. The beginning of the pandemic was associated with dynamic and concomitant disorientation, worsened due to unconfirmed news, primarily spread via social media. We found various transferable solutions described in the literature, such as providing guidance principles for all trainees on deployment, training disruption and its consequences outlined in useful link collections [72] and ‘frequently asked questions’ (FAQ) by journals [43, 87]. Yet, for a better dissemination and to counteract rumors, such publications should be prominently placed on social media [59]. Official authorities should strive for nation-wide consistent approaches that protect the needs of students and residents, including guidance on appropriate deployment, working conditions and academic interests [88]. Nevertheless, it should not be forgotten that despite the undisputed importance of legal aspects, there is also an ethical and moral bound to help [89].

#### (Self-)support and supply

The root for the extensiveness of self-supportive measures partly lies within the dynamic and unprecedentedness of the pandemic itself, where many problems were just realized on the way by those concerned. Initiatives, like supply-boxes are not only directly helpful, but also strengthened the sense of workplace community. Understandably, supply-boxes were associated with an overwhelming positive response [78] and represent an easily transferable solution. The aforementioned student response team to optimize the mobilization of peers was also a huge success and was implemented by several medical schools [77].

The general supportiveness of students and residents was known long before Covid-19. Yet, the current situation rekindled the discussion of whether we may need a longitudinal social justice and advocacy framework in medical school to prepare students even better for such worldwide events [90].

The retrieved articles clearly show that students and residents have an intrinsic ability to adapt creatively to unprecedented scenarios. The implementations of self-supporting measures strengthen the feeling of community within their peer group and hold invaluable implications for faculties, educators and policymakers.

To summarize our findings from the coded subgroups, Table 3 highlights the main concerns and possible solutions.

**Table 3:** Main problems and possible solutions

Main Problem	Possible Solution	Frequent Feedback
Non-Voluntary Deployment	Preceding Interviews to find Appropriate Roles	Communication with Faculties, Educators and Policymakers
Interruption of Training and Career	Clear Legal Guidance and Compensation	
Safety Issues	Provision of Adequate Supervision, Clinical Knowledge, PPE and Testing Capacity	
Well-being	Social Integration and Provision of Mental Health Support	

### Limitations

This review has several limitations. As it focuses on frontline experience from the first 4 months of the pandemic, long-term insights and evidence, especially on complex educational aspects, are not covered. Furthermore, several articles in this review may hold a bias of subjectivity and overrepresented unilateral views. Nevertheless, this review compiles and depicts the early reactions in a pandemic outbreak and may therefore hold valuable insights to learn from.

Another limitation is that our review is solely based on articles written in English language and therefore may lack important content from the non-English literature. Moreover, the search restriction on articles in English language introduces a selection bias and may make certain insights not generalizable; e.g., articles, discussing rights and obligations, were mainly from the United Kingdom and may not apply to foreign legislations.

## Conclusion

The Covid-19 pandemic is not only a challenge but also a chance to change the situation for students and residents for the better. Never has it been easier to raise awareness for trainees’ concerns. Never have the barriers been lower to implement new policies and technology to improve the transition of the young workforce into the field of medicine in times of crisis. Our review of the literature provides thorough implications for faculties, educators and policymakers. Not only to ensure surge capacity, but also to promote the safety and the professional identity formation of students and residents, it is crucial to understand their needs and concerns. Leaders should facilitate close communication with students and residents, value their intrinsic creativeness and regularly evaluate their needs in regards to deployment, knowledge aspects, safety measures, legal concerns and overall well-being.

## Data Availability

Content analysis database is deposited at the DRYAD data repository

## Abbreviations

CA: Content Analysis
Covid-19: Corona Virus Disease 2019
CPR: Cardiopulmonary Resuscitation
ED: Emergency Department
FAQ: Frequently Asked Questions
MeSH: Medical Subject Headings
PPE: Personal Protective Equipment
SARS-CoV-2: Severe Acute Respiratory Syndrome Corona Virus 2
UAE: United Arab Emirates
UK: United Kingdom
USA: United States of America
WHO: World Health Organization

## References

[1] WHO Director-General's opening remarks at the media briefing on COVID-19 - 11 March 2020**.** Available from: https://www.who.int/dg/speeches/detail/who-director-general-s-opening-remarks-at-the-media-briefing-on-covid-19%2D%2D-11-march-2020 [Retrieved 06 June 2020].

[2] Chen PT, Huang YC, Cheng HW, Wang CC, Chan CY, Chan KH, Kuo CD (2009). New simulation-based airway management training program for junior physicians: advanced airway life support. Med Teach.

[3] Wong TW, Tam WW (2005). Handwashing practice and the use of personal protective equipment among medical students after the SARS epidemic in Hong Kong. Am J Infect Control.

[4] Honigsbaum M (2018). Spanish influenza redux: revisiting the mother of all pandemics. Lancet (London, England).

[5] Pneumonia of unknown cause – China. Available from: https://www.who.int/csr/don/05-january-2020-pneumonia-of-unkown-cause-china/en/ [Retrieved 06 June 2020].

[6] SARS-CoV-2 references. Available from: https://zika.ispm.unibe.ch/assets/data/pub/ncov/ [Retrieved 06 June 2020].

[7] Gaber DA, Shehata MH, Amin HAA (2020). Online team-based learning sessions as interactive methodologies during the pandemic. Med Educ.

[8] Alvin MD, George E, Deng F, Warhadpande S, Lee SI (2020). The Impact of COVID-19 on Radiology Trainees. Radiology.

[9] Ebben S, Hussain RA, Miloro M, Callahan N (2020). The UIC COVID coverage protocol: a technical note for pandemic Oral and maxillofacial surgery call coverage. J Oral Maxillofac Surg.

[10] Eichberg DG, Shah AH, Luther EM, Menendez I, Jimenez A, Perez-Dickens M, O’Phelan KH, Ivan ME, Komotar RJ, Levi AD (2020). Letter: academic neurosurgery department response to COVID-19 pandemic: the University of Miami/Jackson memorial hospital model. Neurosurgery.

[11] Armstrong A, Jeevaratnam J, Murphy G, Pasha M, Tough A, Conway-Jones R, Mifsud RW, Tucker S (2020). A plastic surgery service response to COVID-19 in one of the largest teaching hospitals in Europe. J Plast Reconstr Aesthet Surg.

[12] Weber AC, Henderson F, Santos JM, Spiotta AM (2020). Letter: for whom the bell tolls: overcoming the challenges of the COVID pandemic as a residency program. Neurosurgery.

[13] Bray DP, Stricsek GP, Malcolm J, Gutierrez J, Greven A, Barrow DL, Rodts GE, Gary MF, Refai D (2020). Letter: maintaining neurosurgical resident education and safety during the COVID-19 pandemic. Neurosurgery.

[14] Ammar A, Stock AD, Holland R, Gelfand Y, Altschul D (2020). Managing a specialty service during the COVID-19 crisis: lessons from a new York City health system. Acad Med.

[15] Park JS, El-Sayed IH, Young VN, Pletcher SD (2020). Development of clinical care guidelines for faculty and residents in the era of COVID-19. Head Neck.

[16] Phillips CD, Shatzkes DR, Moonis G, Hsu KA, Doshi A, Filippi CG (2020). From the eye of the storm: multi-institutional practical perspectives on neuroradiology from the COVID-19 outbreak in new York City. AJNR Am J Neuroradiol.

[17] Konda SR, Dankert JF, Merkow D, Lin CC, Kaplan DJ, Haskel JD, Behery O, Crespo A, Ganta A (2020). COVID-19 response in the global epicenter: converting a new York City level 1 orthopedic trauma service into a hybrid orthopedic and medicine COVID-19 management team. J Orthop Trauma.

[18] Iserson KV (2020). Augmenting the disaster healthcare workforce. West J Emerg Med.

[19] Villela EFM, de Oliveira FM, Leite ST, Bollela VR (2020). Student engagement in a public health initiative in response to COVID-19. Med Educ.

[20] Anna Harvey: Covid-19—Medical students face disruption and uncertainty. Available from: https://blogs.bmj.com/bmj/2020/03/16/anna-harvey-covid-19-medical-students-face-disruption-and-uncertainty/ [Retrieved 06 June 2020].

[21] Thomson E, Lovegrove S (2020). ‘Let us help’—why senior medical students are the next step in battling the COVID-19 pandemic. Int J Clin Pract.

[22] Wilson AN, Ravaldi C, Scoullar MJL, Vogel JP, Szabo RA, Fisher JRW, Homer CSE (2021). Caring for the carers: ensuring the provision of quality maternity care during a global pandemic. Women Birth.

[23] Rasmussen S, Sperling P, Poulsen MS, Emmersen J, Andersen S (2020). Medical students for health-care staff shortages during the COVID-19 pandemic. Lancet (London, England).

[24] Oxford medical students step-up to support fight against Covid-19. Available from: http://www.ox.ac.uk/news/2020-03-23-oxford-medical-students-step-support-fight-against-covid-19 [Retrieved 06 June 2020].

[25] Harvey A (2020). Covid-19: medical students and FY1 doctors to be given early registration to help combat covid-19. BMJ.

[26] NYU offers early graduation to senior med students in bid to fight coronavirus. Available from: www.nypost.com/2020/03/24/nyu-offers-early-graduation-to-senior-med-students-in-bid-to-fight-coronavirus/ [Retrieved 06 June 2020].

[27] Schwarzkopf R, Maher NA, Slover JD, Strauss EJ, Bosco JA, Zuckerman JD (2020). The response of an orthopedic department and specialty Hospital at the Epicenter of a pandemic: the NYU Langone health experience. J Arthroplast.

[28] Mahase E (2020). Covid-19: medical students to be employed by NHS. BMJ.

[29] Basky G (2020). All hands on deck as cases of COVID-19 surge. Can Med Assoc J.

[30] Harvey A (2020). Covid-19: medical students should not work outside their competency, says BMA. BMJ.

[31] Rusconi A (2006). Leaving the parental home in West-Germany and Italy. Opportunities and constraints.

[32] Kalet AL, Jotterand F, Muntz M, Thapa B, Campbell B (2020). Hearing the call of duty: what we must do to allow medical students to respond to the COVID-19 pandemic. WMJ.

[33] Stetson GV, Kryzhanovskaya IV, Lomen-Hoerth C, Hauer KE (2020). Professional identity formation in disorienting times. Med Educ.

[34] Saibene AM, Allevi F, Biglioli F, Felisati G (2020). Role and Management of a Head and Neck Department during the COVID-19 outbreak in Lombardy. Otolaryngol Head Neck Surg.

[35] Ross SW, Lauer CW, Miles WS, Green JM, Christmas AB, May AK, Matthews BD (2020). Maximizing the Calm before the Storm: Tiered Surgical Response Plan for Novel Coronavirus (COVID-19). J Am Coll Surg.

[36] Niska RW (2005). BCW: bioterrorism and mass casualty preparedness in hospitals: United States, 2003. Adv Data.

[37] Martin SD, Bush AC, Lynch JA (2006). A national survey of terrorism preparedness training among pediatric, family practice, and emergency medicine programs. Pediatrics.

[38] Dinh TT, Halasz LM, Ford E, Rengan R (2020). Radiation therapy in King County, Washington during the COVID-19 pandemic: balancing patient care, transmission mitigation, and resident training. Adv Radiat Oncol.

[39] Chong A, Kagetsu NJ, Yen A, Cooke EA (2020). Radiology residency preparedness and response to the COVID-19 pandemic. Acad Radiol.

[40] Eva KW (2020). Strange days. Med Educ.

[41] Cao W, Fang Z, Hou G, Han M, Xu X, Dong J, Zheng J (2020). The psychological impact of the COVID-19 epidemic on college students in China. Psychiatry Res.

[42] Solomon HV (2020). COVID-19 checklist: mask, gloves, and video chatting with grandpa. Psychiatry Res.

[43] Rimmer A (2020). Covid-19: what do trainees need to know?. BMJ.

[44] Warhadpande S, Khaja MS, Sabri SS (2020). The impact of COVID-19 on interventional radiology training programs: what you need to know. Acad Radiol.

[45] Clark VE (2020). Editorial**.** Impact of COVID-19 on neurosurgery resident research training. J Neurosurg.

[46] Harrington RA, Elkind MSV, Benjamin IJ (2020). Protecting medical trainees on the COVID-19 frontlines saves us all. Circulation.

[47] Wong CS, Tay WC, Hap XF, Chia FL (2020). Love in the time of coronavirus: training and service during COVID-19. Singap Med J.

[48] Dyer GSM, Lipa SA (2020). What’s important: COVID-19—helpers, not heroes. J Bone Joint Surg Am.

[49] Tabari P, Amini M, Moosavi M (2020). Lessons learned from COVID-19 epidemic in Iran: the role of medical education. Med Teach.

[50] He K, Stolarski A, Whang E, Kristo G (2020). Addressing general surgery Residents' concerns in the early phase of the COVID-19 pandemic. J Surg Educ.

[51] Liang ZC, Ooi SBS (2020). COVID-19: a Singapore orthopedic Resident’s musings in the emergency department. Acad Emerg Med.

[52] Ross JE (2020). Resident response during pandemic: this is our time. Ann Intern Med.

[53] Gallagher TH, Schleyer AM (2020). “We signed up for this!” — student and trainee responses to the Covid-19 pandemic. N Engl J Med.

[54] Gautam M, Kaur M, Mahr G (2020). COVID-19-associated psychiatric symptoms in health care workers: viewpoint from internal medicine and psychiatry residents. Psychosomatics.

[55] Grant-Kels JM (2020). Invited response to the comment on “dermatology residents and the care of COVID-19 patients”. J Am Acad Dermatol.

[56] Patel B (2020). Comment on “dermatology residents and the care of COVID-19 patients”. J Am Acad Dermatol.

[57] Culp BM, Frisch NB (2020). COVID-19 impact on Young arthroplasty surgeons. J Arthroplast.

[58] Menon A, Klein EJ, Kollars K, Kleinhenz ALW (2020). Medical students are not essential workers: examining institutional responsibility during the COVID-19 pandemic. Acad Med.

[59] Agarwal V, Gupta L, Davalbhakta S, Misra D, Agarwal V, Goel A. Undergraduate medical students in India are underprepared to be the young-taskforce against Covid-19 amid prevalent fears. medRxiv 2020.04.11.20061333. 10.1101/2020.04.11.20061333

[60] Khan S, Khan M, Maqsood K, Hussain T, Ul-Huda N, Zeeshan M (2020). Is Pakistan prepared for the COVID-19 epidemic? A questionnaire-based survey. J Med Virol.

[61] Bhagavathula AS, Aldhaleei WA, Rahmani J, Mahabadi MA, Bandari DK (2020). Knowledge and perceptions of COVID-19 among health care workers: cross-sectional study. JMIR Public Health Surveill.

[62] Burhan Dost EK, Terzi Ö, Bilgin S, Ustun YB, Arslan HN (2020). Attitudes of Anesthesiology Specialists and Residents toward Patients Infected with the Novel Coronavirus (COVID-19): A National Survey Study. Surg Infect.

[63] Taghrir MH, Borazjani R, Shiraly R (2020). COVID-19 and Iranian medical students; a survey on their related-knowledge, preventive behaviors and risk perception. Arch Iran Med March.

[64] Alzoubi H, Alnawaiseh N, Al-Mnayyis A, Lubad M, Aqel A, Al-Shagahin H (2020). COVID-19-knowledge, attitude and practice among medical and non-medical university students in Jordan. J Pure Appl Microbiol.

[65] DeFilippis EM, Ranard LS, Berg DD (2020). Cardiopulmonary resuscitation during the COVID-19 pandemic: a view from trainees on the frontline. Circulation.

[66] Gilbert A (2020). Trainees and COVID-19: a call to arms. Am J Forensic Med Pathol.

[67] Poonia SK, Rajasekaran K (2020). Information overload: a method to share updates among frontline staff during the COVID-19 pandemic. Otolaryngol Head Neck Surg.

[68] Kim H (2020). Outbreak of novel coronavirus (COVID-19): what is the role of radiologists?. Eur Radiol.

[69] Wujtewicz M, Dylczyk-Sommer A, Aszkiełowicz A, Zdanowski S, Piwowarczyk S, Owczuk R (2020). COVID-19 - what should anaethesiologists and intensivists know about it?. Anaesthesiol Intensive Ther.

[70] Escalera-Antezana JP, Cerruto-Zelaya PE, Apaza-Huasco M, Miranda-Rojas SH, Flores-Cárdenas CA, Rivera-Zabala L, Olmos-Machicado JR, Alvarez-Amaya V, Acevedo-López D, Valencia-Gallego V, López-Echeverri C, Vallejo-Atehortua E, González-Patiño V, Vásquez-Castañeda DL, García-Zuluaga LM, Cortés-Bonilla I, López-Bueno I, Villamil-Gómez WE, Otero-Florez JM, Toscano-Lobo CE, González GM, Díaz-Guio DA, Rodríguez-Morales AJ (2020). Healthcare workers' and students' knowledge regarding the transmission, epidemiology and symptoms of COVID-19 in 41 cities of Bolivia and Colombia. Travel Med Infect Dis.

[71] Christensen L, Rasmussen CS, Benfield T, Franc JM (2020). A randomized trial of instructor-led training versus video lesson in training health care providers in proper donning and doffing of personal protective equipment. Disaster Med Public Health Prep.

[72] Rimmer A (2020). Trainees and covid-19: your questions answered. BMJ.

[73] Rimmer A (2020). Covid-19: trainees will not move jobs in April. BMJ.

[74] Guidance on medical students’ clinical participation: Effective immediately. Available from: https://lcme.org/wp-content/uploads/filebase/March-17-2020-Guidance-on-Mediical-Students-Clinical-Participation.pdf [Retrieved 06 June 2020].

[75] Covid-19: medical students requested to work in the NHS. Available from: https://beta.bma.org.uk/advice-and-support/covid-19/your-contract/covid-19-medical-students-requested-to-work-in-the-nhs [Retrieved 06 June 2020].

[76] Stokes DC (2020). Senior medical students in the COVID-19 response: an opportunity to be proactive. Acad Emerg Med.

[77] Soled D, Goel S, Barry D, Erfani P, Joseph N, Kochis M, Uppal N, Velasquez D, Vora K, Scott KW (2020). Medical student mobilization during a crisis: lessons from a COVID-19 medical student response team. Acad Med.

[78] Rimmer A (2020). Covid-19: junior doctor calls on colleagues to gather supplies for staff working long hours. BMJ.

[79] Rakowsky S, Flashner BM, Doolin J, Reese Z, Shpilsky J, Yang S, Smith CC, Graham K (2020). Five questions for residency leadership in the time of COVID-19: reflections of chief medical residents from an internal medicine program. Acad Med.

[80] Information for medical students. Available from: https://www.gmc-uk.org/news/news-archive/coronavirus-information-and-advice/information-for-medical-students [Retrieved 06 June 2020].

[81] Villa A, Sankar V, Shiboski C (2021). Tele (oral)medicine: a new approach during the COVID-19 crisis. Oral Dis.

[82] Dieckmann P, Torgeirsen K, Qvindesland SA, Thomas L, Bushell V, Langli Ersdal H (2020). The use of simulation to prepare and improve responses to infectious disease outbreaks like COVID-19: practical tips and resources from Norway, Denmark, and the UK. Adv Simul.

[83] Park J, Rhim HC (2020). Consequences of coronavirus disease 2019 on international medical graduates and students applying to residencies in the United States. Korean J Med Educ.

[84] Abdessater M, Rouprêt M, Misrai V, Matillon X, Gondran-Tellier B, Freton L, Vallée M, Dominique I, Felber M, Khene ZE, Fortier E, Lannes F, Michiels C, Grevez T, Szabla N, Boustany J, Bardet F, Kaulanjan K, Seizilles de Mazancourt E, Ploussard G, Pinar U, Pradere B, Association Française des Urologues en Formation (AFUF) (2020). COVID19 pandemic impacts on anxiety of French urologist in training: outcomes from a national survey. Prog Urol.

[85] Ullah R, Amin S (2020). The psychological impact of COVID-19 on medical students [letter]. Psychiatry Res.

[86] Rimmer A (2020). How can I cope with redeployment?. BMJ.

[87] Rimmer A (2020). Covid-19: health education England shares advice for trainees. BMJ.

[88] Representatives of the STARSurg Collaborative EC, and TASMAN Collaborative (2020). Medical student involvement in the COVID-19 response. Lancet (London, England).

[89] Stoj VJ, Grant-Kels JM (2020). Dermatology residents and the care of patients with coronavirus disease 2019 (COVID-19). J Am Acad Dermatol.

[90] Cantave M, Perlson J, Lewis C, Byers B (2020). COVID-19 reveals why we need physician-advocates now. Acad Med.

